# Risk factors for development of diabetic foot ulcer disease in two large contemporary UK cohorts

**DOI:** 10.1111/dom.16519

**Published:** 2025-06-24

**Authors:** Safoora Gharibzadeh, Jiyoung Lee, Patrick Highton, Nicola Greenlaw, Clare Gillies, Francesco Zaccardi, Alan Brennan, Daniel John Pollard, Jonathan Valabhji, Frances Game, Bethany Stanley, Graham Leese, Laura Gray, Solomon Tesfaye, David Webb, Sarah Wild, Sharmin Shabnam, Melanie Davies, Kamlesh Khunti, John Petrie, Edward Gregg

**Affiliations:** ^1^ Leicester Real World Evidence Unit, Diabetes Research Centre, Leicester General Hospital, University of Leicester, Leicester UK; ^2^ Robertson Centre for Biostatistics, School of Health and Wellbeing, College of Medical, Veterinary and Life Sciences University of Glasgow Glasgow UK; ^3^ Leicester UK; ^4^ National Institute for Health and Care Research Applied Research Collaboration East Midlands, Leicester, UK University of Glasgow UK; ^5^ SCHARR, Sheffield Centre for Health and Related Research, Division of Population Health, School of Medicine and Population Health University of Sheffield Sheffield UK; ^6^ Department of Metabolism, Digestion and Reproduction, Faculty of Medicine Chelsea and Westminster Hospital Campus, Imperial College London London UK; ^7^ Derby UK; ^8^ NHS Tayside, Scotland Dundee, UK Scotland UK; ^9^ Department of Population Health Sciences University of Leicester Leicester UK; ^10^ Division of Clinical Medicine University of Sheffield Sheffield UK; ^11^ Diabetes Research Unit Sheffield Teaching Hospitals NHS Foundation Trust Sheffield UK; ^12^ Diabetes Research Centre, College of Life Sciences University of Leicester Leicester UK; ^13^ Centre for Population Health Sciences Usher Institute, University of Edinburgh, University of Leicester Leicester UK; ^14^ National Institute for Health Research Leicester Biomedical Research Centre Leicester UK; ^15^ School of Health and Wellbeing, College of Medical, Veterinary and Life Sciences, University of Glasgow, Clarice Pears Building, Level 5 90 Byres Road Glasgow UK; ^16^ School of Population Health RCSI University of Medicine and Health Sciences Dublin Ireland

**Keywords:** cardiovascular disease, diabetes complications, real‐world evidence, type 1 diabetes, type 2 diabetes

## Abstract

**Aims:**

Diabetic foot ulcer disease (DFUD) is common, life‐changing and associated with a lower 5‐Year survival rate than many cancers. However, the risk factors for DFUD have generally been identified in small, single‐centre, clinic‐based studies, many of which are cross‐sectional. This study aims to assess the incidence of DFUD and its related risk factors in two large, contemporary UK cohorts.

**Materials and Methods:**

We investigated common sociodemographic and clinical factors affecting the incidence rates of DFUD in two large representative independent cohorts of people with diabetes in England (CPRD, *n* = 131 042) and Scotland (Scottish Diabetes Research Network‐National Diabetes Dataset [SDRN‐NDS] *n* = 260 748). The methods of case ascertainment differed between the two cohorts: in England, both primary and secondary care data were used, whereas in Scotland, secondary care and foot clinic data were used.

**Results and Conclusions:**

In the English cohort, 4.7% developed DFUD over a median of 4.3years (incidence rate 9.0[95%CI: 8.8–9.2] per 1000 person‐years) follow‐up; in the Scottish cohort, the equivalent figure was 2.9% over a median of 6.3 years (incidence rate 4.4 [95% CI: 4.3–4.5] per 1000 person‐years). Despite different methods of case ascertainment, multivariable analysis in both populations indicated that those who developed DFUD were more likely to be older, male, smokers, of White ethnicity, with higher systolic blood pressure and baseline HbA1c. These findings provide a robust evidence base for identifying people with diabetes at risk of DFUD for targeted efforts for prevention.

## 1 INTRODUCTION

Diabetic foot ulcer disease (DFUD) is a common, life‐changing complication of poor glycaemic control,^1^ preceding 80% of diabetes‐related lower limb amputations and carrying a high mortality risk. Annually, DFUD affects ~18.6 million people worldwide, including 1.6 million in the United States,^2^ with an estimated 2% of individuals with diabetes in England developing a new ulcer each year.^3^ This results in 50–60 000 active DFUD cases and a UK incidence of ~40–50 000 annually.^4^ A 2017 meta‐analysis of 67 studies estimated a global DFUD prevalence of 6.3%, with regional variation.^5^

In those with DFUD, five‐year survival is worse than many cancers at 40%–50%,^6^, ^7^ declining to 30% post‐amputation.^8^, ^9^ The economic burden is substantial, with DFUD costing the NHS more annually than breast, prostate and lung cancers combined,^3^ and accounting for a third of the $237 billion direct diabetes costs in the United States in 2017.^10^

Previous systematic reviews have identified multiple predictive factors for diabetic foot ulceration, highlighting the complexity of risk assessment in this condition.^11^ In a landmark prospective study, the Seattle Diabetic Foot Study demonstrated that increased plantar pressure, reduced ankle‐arm index, visual impairment and poor glucose control were independent predictors of foot ulceration in patients with diabetes.^12^

DFUD incidence is influenced by clinical, sociodemographic and care‐related factors. Key clinical risks include neuropathy, peripheral vascular disease, poor glycaemic control, long diabetes duration,^11^ retinopathy and nephropathy^13^ and possibly obesity.^14^ Clinical risk factors for DFUD‐related complications (e.g., further ulceration, gangrene, amputation) are similar, but also include cerebral vascular disease, coronary artery disease, hypertension and other cardiovascular risk factors.^15^, ^16^ Age, deprivation and geography are major sociodemographic drivers of DFUD.^8^, ^17^, ^18^, ^19^ Ethnicity may also play a role, with limited evidence suggesting lower DFUD risk in UK South Asians due to reduced peripheral arterial disease (PAD), neuropathy, insulin use and foot deformities.^20^

In order to better inform more accurate risk stratification and prevention of DFUD and its complications in people living with diabetes, a thorough understanding of the full range of drivers of DFUD incidence and risk factors is vital. In this study, we took a harmonized approach to the analysis of two major population‐based cohorts, the UK Clinical Practice Research Datalink (CPRD GOLD) and the Scottish Diabetes Research Network‐National Diabetes Dataset (SDRN‐NDS), with the aim of providing a comprehensive examination of the incidence and risk factors of DFUD in people with diabetes.

## 2 MATERIALS AND METHODS

We utilized two major population‐based data systems from England (2000–2021) and Scotland (2007–2021) to construct dynamic cohorts for retrospective longitudinal studies. The study included adults (≥18 years) with diagnosed diabetes. Reporting followed the RECORD guidelines (checklist in Supporting Information).^21^

### 2.1 Cohorts

The Clinical Practice Research Datalink (CPRD) GOLD contains de‐identified primary care records from 674 UK general practices, covering over 11.3 million patients (~6.9% of the population) and broadly representative of the national demographics. More than half of CPRD patients are eligible for linkage to additional datasets, including hospital records, cancer registries and mortality data. In England, 75% of practices (58% of all UK CPRD practices) participate in the CPRD linkage scheme, enabling connections to Hospital Episode Statistics, Office for National Statistics mortality data, and the Index of Multiple Deprivation.^22^ The use of CPRD data was approved by the CPRD Independent Scientific Advisory Committee (ISAC protocol no. 21_001680).

In Scotland, the Scottish Care Information (SCI)‐Diabetes system covers >99% of diagnosed diabetes cases across all primary care practices and hospital clinics, tracking ~500 000 individuals since 2006 in a population of 5.5 million. SCI‐Diabetes data are linked to hospital and mortality records via the Scottish Diabetes Research Network – National Dataset (NDS). Data linkage and research use were approved by the Public Benefit and Privacy Panel for Health and Social Care (1617‐0147) and the West of Scotland Research Ethics Committee (21/WS/0047). The dataset used for this analysis were released in December 2022 and accessed via the DIAB‐EPI platform.

### 2.2 Analytic Sample

The CPRD analytic sample included individuals aged ≥18 with a first recorded diagnosis of Type 1 or Type 2 diabetes between 01/01/2000 and 01/12/2021. Exclusions applied to those without Hospital Episode Statistics (HES)/Office for National Statistics (ONS) linkage, those who died before their diabetes diagnosis, individuals with prevalent DFUD (in CPRD or HES) before diagnosis, and those diagnosed after the ONS end date (Figure S1).

The Scottish cohort included individuals aged ≥18 with a first diabetes diagnosis between 01/01/2007 and 29/03/2021, residing in a Scottish Health Board region. As SDRN‐NDS data were complete from 01/01/2006, a 2007 start allowed a 12‐month baseline period for defining prevalent conditions and medication history. Those with prior DFUD records in primary or secondary care were excluded.

### 2.3 Outcome

Case ascertainment varied between datasets: in England, DFUD events were identified using primary care data (including foot clinic referrals) and HES ICD‐10 codes, while in Scotland, they were identified via SMR01 ICD‐10 codes and SCI‐Diabetes foot clinic data, covering inpatient and day case records. Follow‐up spanned from diabetes diagnosis to the earliest of DFUD occurrence, HES‐ONS linkage end (March 29, 2021), death, transfer‐out date (England only) or last practice data collection (England only).

### 2.4 Covariates

In England, demographic data and laboratory measurements were obtained from CPRD and HES. Ethnicity was sourced from HES admitted patient care records, with CPRD used if unavailable. Deprivation was assessed using the Index of Multiple Deprivation (IMD2019), calculated by the ONS based on seven weighted domains (income, employment, education, health, crime, housing barriers and living environment) and categorized into quintiles (1 = least deprived, 5 = most deprived).^23^

In Scotland, SCI‐Diabetes provides sociodemographic data (including ethnicity and deprivation) and laboratory measurements. Deprivation was assessed using SIMD 2016 or, if unavailable, SIMD 2020, categorized into quintiles (1 = most deprived, 5 = least deprived). Prevalent clinical and comorbid conditions were identified through hospital admissions, the Scottish Renal Registry, the Scottish Cancer Registry and other relevant datasets.

We analysed sociodemographic predictors, clinical and behavioural risk factors and prevalent conditions, including smoking, body mass index (BMI), systolic blood pressure (SBP), diastolic blood pressure (DBP), serum creatinine, eGFR, HbA1c, Non‐HDL‐C. Comorbidities included cancer, COPD, dementia, depression, ESKD, heart failure, hypertension, MI, neurological disorders, retinopathy and stroke. A binary multiple long‐term condition (MLTC) variable was defined for individuals with two or more chronic conditions.^24^ All laboratory measurements were collected within 12 months before the index date, using the last available data.

Prevalent cardiovascular disease (CVD) was defined as a previous history of either MI or stroke. BMI was categorized according to the WHO definition as follows: less than 24.99 kg/m^2^, between 25 and 29.99 kg/m^2^ and greater than 30 kg/m^2.^ ^25^ HbA1c was categorized as <7%, 7% to <8%, 8% to <9% and ≥9%.

### 2.5 Statistical analysis

Each cohort was analysed separately, using the same agreed pre‐specified analytical approach as follows.

Continuous clinical risk factors were assessed using the Shapiro–Wilk test and graphical analysis. Skewed biomarkers were summarized with medians and IQRs, with group differences tested using the Mann–Whitney U test. Categorical variables were presented as counts and percentages, and group differences assessed using the Chi‐square test. A purposeful variable selection approach was applied: univariate analysis identified statistically significant variables for multivariate analysis, using the Wald test with a *p*‐value cut‐off of 0.25. Non‐significant covariates were removed (alpha = 0.1, 20% change for confounding), and significant covariates and confounders were retained. Non‐selected variables were added back iteratively if significant at the 0.1 level, yielding a preliminary main effects model. This process yields a preliminary main effects model.^26^

To evaluate the association between the explanatory variables and the incidence of DFUD, a Royston‐Parmar proportional hazards model was employed.^27^ A delayed entry structure was used (i.e., left truncation), with age as the time scale. This approach ensured that individuals were considered at risk from the age they were diagnosed with diabetes. The Akaike information criterion (AIC) was applied to select the optimum number of spline knots in the Royston‐Parmar models.^27^

We complemented complete case analyses with multiple imputation. The RBtest package in R was used to test the missing data mechanism (MCAR vs. MAR).^28^ Random forest imputation, chosen for its ability to handle nonlinear relationships, interactions and large datasets like EHRs, reduces overfitting by combining outputs from multiple regression trees.^29^ Shah et al. (2014) showed it outperforms parametric methods in efficiency and precision, making it ideal for this study.^30^

A sensitivity analysis was conducted on individuals diagnosed with diabetes from 2007 onwards, reflecting improved data quality after the Quality and Outcome Framework (QOF) update in 2006^31^ and aligning with the Scottish cohort. Another analysis used 2010 as a temporal divider, marking FDA approval of Sodium‐glucose cotransporter‐2 (SGLT2) inhibitors, a class of medications known to significantly alter the quality of treatment for patients with diabetes.^32^

Analyses were conducted in Stata (StataCorp. 2023. Stata Statistical Software: Release 18. College Station, TX: StataCorp LLC.) and R version 4.1.2 (R Core Team (2023). _R: A Language and Environment for Statistical Computing_. R Foundation for Statistical Computing, Vienna, Austria. https://www.R-project.org/.) and results are reported with 95% CIs. In line with *The Lancet* journal's guidelines, P‐values were reported to two significant figures, except when *p* < 0.0001.

The analysis of CPRD data was conducted using the ALICE High Performance Computing Facility at the University of Leicester.

## 3 RESULTS

### 3.1 Descriptive and Univariate Results

#### 3.1.1 England

Among 131 042 individuals with diabetes (mean age 59.8 ± 15.8 years), 50.1% were male, 88.5% were White and 40.5% had MLTC. Over a median follow‐up of 4.3 years [IQR: 1.8–7.8], 4.8% (*n* = 6234) developed DFUD. Compared to those without DFUD, patients with DFUD were older (65.5 ± 13.9 vs. 59.5 ± 15.8), more often male (54.9%), current smokers (24.9% vs. 23.3%) and White (95.9% vs. 88.1%), with fewer from South Asian (1.5% vs. 3.9%) or Black (0.9% vs. 1.9%) backgrounds. They also had higher pre‐diagnosis HbA1c levels (56.3 vs. 51.9 mmol/mol) and more long‐term conditions, except for depression (Table 2).

#### 3.1.2 Scotland

Of 260 748 individuals with diabetes (mean age 59.4 ± 13.9 years), 56.7% were male, 90.7% were White and 25.7% (*n* = 67 117) had MLTC (Tables 1 and 2). Over a median follow‐up of 6.3 years [IQR: 3.2–9.8], 2.9% (*n* = 7508) developed DFUD. Compared to those without DFUD, patients with DFUD were older (63.9 ± 14.2 vs. 59.3 ± 13.9 years), more often male (61.8% vs. 56.6%), current smokers (26.1% vs. 21.9%) and White (95.1% vs. 90.5%), with fewer from South Asian (1.1% vs. 3.4%) or Black (0.2% vs. 0.9%) backgrounds. They also had higher pre‐diagnosis HbA1c levels (61 vs. 55 mmol/mol).

**TABLE 1:** Characteristics of the cohort at index date.

	Total population	
	CPRD GOLD (*N* = 131 042)	SDRN‐NDS (*N* = 260 748)
Age (years)	59.8 (15.8)	59.4 (13.9)
Sex		
Male	65 664 (50.1%)	147 894 (56.7%)
Female	65 378 (49.9%)	112 854 (43.3%)
Ethnicity		
White	110 147 (88.5%)	184 150 (90.7%)
South Asian	4749 (3.8%)	6771 (3.3%)
Black	2297 (1.8%)	1760 (0.9%)
Mixed‐Other	7268 (5.8%)	10 435 (5.1%)
Smoking Status		
Current Smoker	22 943 (23.4%)	53 373 (22.0%)
Ex‐smoker	39 780 (40.6%)	91 552 (37.8%)
Never Smoker	35 291 (36.0%)	97 247 (40.2%)
IMD (Quintile)		
Least Deprived	24 313 (18.6%)	‐
2	25 749 (19.7%)	‐
3	28 316 (21.6%)	‐
4	26 994 (20.6%)	‐
Most Deprived	25 607 (19.6%)	‐
SMID (quintile)		
Most Deprived	‐	63 757 (24.6%)
2	‐	59 968 (23.1%)
3	‐	52 714 (20.3%)
4	‐	46 794 (18.0%)
Least Deprived	‐	36 196 (14.0%)
BMI status		
<25 kg/m^2^	14 743 (11.2%)	11 918 (8.7%)
25–30 kg/m^2^	36 979 (28.2%)	34 576 (25.2%)
≥30 kg/m^2^	73 539 (56.1%)	90 817 (66.1%)
BMI (kg/m^2^)	31.4 (27.6–36.2)	32.4 (28.5–37.2)
SBP (mmHg)	80.8 (11.4)	138.0 (17.2)
DBP (mmHg)	138.1 (18.9)	80.8 (10.8)
Creatinine(μmol/L)	81.0 (69.0–95.0)	78.0 (67.0–91.0)
eGFR (ml/min/1.73 m^2^)	77.0 (61.0–90.0)	78.3 (65.7–92.4)
Pre‐diagnosis HbA1c (%)	6.9 (6.3–8.3)	7.2 (6.6–9.2)
Pre‐diagnosis HbA1c(mmol/ml)	52.0 (45.4–67.2)	55.0 (49.0–77.0)
Non‐HDL (mmol/L)	3.8 (3.1–4.7)	3.9 (3.1–4.7)

*Note*: Data are presented as mean (SD) or median (IQR) for continuous measures, and n (%) for categorical measures.

Abbreviations: BMI, body mass index; DBP, Diastolic Blood Pressure; eGFR, estimated Glomerular Filtration Rate; HbA1c, glycated haemoglobin; IMD, Index of Multiple Deprivation; SBP, Systolic Blood Pressure; SMID, Scottish Index of Multiple Deprivation.

**TABLE 2:** Prevalent conditions status at the time of diagnosis of DM (index date) by incident DFUD.

	CPRD			SDRN‐NDS		
	Total	Non‐DFUD	DFUD	Total	Non‐DFUD	DFUD
	*N* = 131 042	*N* = 124 808	*N* = 6234	*N* = 260 748	*N* = 253 240	*N* = 7508
Atrial fibrillation	9142 (7.0%)	8336 (6.7%)	806 (12.9%)	12 770 (4.9%)	12 023 (4.7%)	747 (9.9%)
Cancer	12 993 (9.9%)	12 354 (9.9%)	639 (10.3%)	24 924 (9.6%)	24 121 (9.5%)	803 (10.7%)
COPD	8919 (6.8%)	8340 (6.7%)	579 (9.3%)	10 324 (4.0%)	9876 (3.9%)	448 (6.0%)
CKD	8318 (6.3%)	7798 (6.2%)	520 (8.3%)	63 897 (24.5%)	61 580 (24.3%)	2317 (30.9%)
Dementia	1456 (1.1%)	1387 (1.1%)	69 (1.1%)	1095 (0.4%)	1044 (0.4%)	51 (0.7%)
Depression	31 215 (23.8%)	29 811 (23.9%)	1404 (22.5%)	8218 (3.2%)	7945 (3.1%)	273 (3.6%)
ESRD	1058 (0.8%)	967 (0.8%)	91 (1.5%)	2012 (0.8%)	1881 (0.7%)	131 (1.7%)
Heart Failure	5352 (4.1%)	4855 (3.9%)	497 (8.0%)	2568 (1.0%)	2369 (0.9%)	199 (2.7%)
Hypertension	59 973 (45.8%)	56 340 (45.1%)	3633 (58.3%)	43 028 (16.5%)	41 403 (16.3%)	1625 (21.6%)
MI	8122 (6.2%)	7624 (6.1%)	498 (8.0%)	15 519 (6.0%)	14 964 (5.9%)	555 (7.4%)
Neuropathy	679 (0.5%)	608 (0.5%)	71 (1.1%)	67 (0.0%)	^b^ (^b^%)	^b^ (^b^%)
Peripheral arterial disease	2794 (2.1%)	2406 (1.9%)	388 (6.2%)	4266 (1.6%)	3874 (1.5%)	392 (5.2%)
Retinopathy	1132 (0.9%)	1057 (0.8%)	75 (1.2%)	155 (0.1%)	^b^ (^b^%)	^b^ (^b^%)
Stroke	8758 (6.7%)	8126 (6.5%)	632 (10.1%)	11 327 (4.3%)	10 822 (4.3%)	505 (6.7%)
Cardiovascular diseases	21 348 (16.3%)	19 867 (15.9%)	1481 (23.8%)	45 220 (17.3%)	43 250 (17.1%)	1970 (26.2%)
Multiple long‐term conditions^a^	53 025 (40.5%)	49 782 (39.9%)	3243 (52.0%)	67 117 (25.7%)	64 404 (25.4%)	2713 (36.1%)

Abbreviations: COPD, Chronic obstructive pulmonary disease; CKD, Chronic kidney disease; ESRD, End‐Stage Renal Disease; MI, Myocardial Infarction.

^a^ Having two or more long‐term conditions.

^b^ Categories with a frequency below 10.

The medication history at the index date and 180 days prior for individuals both with and without DFUD is presented in Table S2. It was observed that neuropathy medication constituted the most prevalent category of medications in Scotland, while Metformin was predominantly used in England.

### 3.2 Incidence and Risk Factors for DFUDs

DFUD crude incidence rates differed between countries: 9.0 [95% CI: 8.8–9.2] per 1000 person‐years in England versus 4.4 [95% CI: 4.3–4.5] in Scotland. Both countries showed similar gender patterns, with males having about 23% higher rates than females (IRR: 1.2). In both nations, patients with MLTCs had double the rate of those without (England: 12.7 vs. 6.8; Scotland: 7.0 vs. 3.6 per 1000 person‐years). Age‐related increases were observed in both countries, with rates lowest in those under 40 (England: 3.7 [95% CI: 3.2–4.1]; Scotland: 2.5 [95% CI: 2.3–2.7]) and highest in those over 85 years (England: 23.8 [95% CI: 21.6–26.2]; Scotland: 16.6 [95% CI: 14.8–18.3]). The incidence of DFUD in various population subgroups, along with corresponding 95% confidence intervals (CI), is detailed in Table S3 of the Supporting Information. Annual incidence rates in both datasets exhibited certain fluctuations, with the trend observed in Scotland displaying less variability. In both nations, the concluding incidence was lower than that recorded at the commencement of the study period (Table S [7]; Graphs[S2] and [S3]).

### 3.3 Multivariable Findings in England and Scotland

Both datasets used the same statistical algorithm to determine the final set of variables included in the model. The final models using CPRD GOLD and SDRN‐NDS resulted in different sets of variables.

Living in the most deprived areas increased DFUD risk compared to the least deprived areas, with a higher impact in England (hazard ratios (HR): 2.4 [95% CI: 2.0–2.9]) than in Scotland (HR: 1.4 [95% CI: 1.2–1.8]). Pre‐diagnosis HbA1c levels above 8% showed increased risk compared to levels below 7%: for 8%–9%, England HR: 2.0, Scotland HR: 1.4; for ≥9%, England HR: 2.1, Scotland HR: 2.0. PAD history doubled DFUD risk in both countries (England HR: 2.0 [1.6–2.6]; Scotland HR: 2.6 [2.0–3.3]). Current smoking increased risk only in Scotland (HR: 1.5 [1.3–1.8]). Atrial fibrillation history increased risk similarly in both countries (England HR: 1.5 [1.3–1.8]; Scotland HR: 1.7 [1.4–2.1]) (Table 3).

**TABLE 3:** Hazard ratios (HR) of potential risk factors for diabetic foot ulcer disease (DFUD) in complete case analysis.

	CPRD		SDRN‐NDS	
	HR (95% CI)	*p*‐value	HR (95% CI)	*p*‐value
Age at diagnosis (years)	1.02 (1.02, 1.03)	<0.0001	1.04 (1.04, 1.05)	<0.0001
Sex				
Female versus Male			0.87 (0.76, 0.98)	0.02
Ethnicity				
Ethnic Minority versus White	0.46 (0.36, 0.58)	<0.0001	‐	‐
Index of Multiple deprivation				
Least deprived(reference)	‐	‐	‐	‐
2	1.32 (1.08, 1.62)	<0.0001	‐	‐
3	1.58 (1.31, 1.92)	<0.0001	‐	‐
4	1.43 (1.17, 1.75)	<0.0001	‐	‐
Most deprived	2.43 (2.03, 2.92)	<0.0001	‐	‐
SIMD				
Most deprived	‐	‐	1.44 (1.17, 1.79)	0.00076
2	‐	‐	1.36 (1.09, 1.68)	0.0056
3	‐	‐	1.3 (1.05, 1.62)	0.02
4	‐	‐	1.21 (0.96, 1.52)	0.10
Least deprived (reference)	‐	‐	‐	‐
Smoking				
Current smoker			1.58 (1.34, 1.86)	<0.0001
Ex‐smoker			1.02 (0.88, 1.17)	0.83
Never smoker(reference)			‐	
Non‐HDL‐C(mmol/L)	0.99 (0.95, 1.04)	0.99	0.91 (0.85, 0.97)	0.0062
HbA1c category				
<7%(reference)	‐		‐	
7%–8%	1.49 (1.28, 1.73)	<0.0001	1.27 (1.09, 1.49)	0.0021
8%–9%	2.01 (1.67, 2.42)	<0.0001	1.36 (1.09, 1.68)	0.0056
≥9%	2.09 (1.81, 2.42)	<0.0001	1.97 (1.69, 2.29)	<0.0001
BMI groups				
<25 kg/m^2^				
25–30 kg/m^2^			0.76 (0.62, 0.94)	0.01
≥30 kg/m^2^			0.79 (0.65, 0.96)	0.02
SBP (mmHg)^a^			1.05 (1, 1.09)	0.04
DBP (mmHg)^a^	1.07 (1.04, 1.10)	<0.0001	0.86 (0.8, 0.93)	<0.0001
eGFR(ml/min/1.73 m^2^)			1.01 (1, 1.01)	0.0044
*Long‐term conditions*				
Peripheral arterial disease	2.08 (1.66, 2.60)	<0.0001	2.63 (2.06, 3.36)	<0.0001
Atrial fibrillation	1.57 (1.32, 1.87)	<0.0001	1.79 (1.48, 2.17)	<0.0001
Heart failure	1.62 (1.31, 2.01)	<0.0001	1.84 (1.3, 2.61)	0.00058
CKD	1.07 (0.91, 1.27)	0.38	1.16 (0.99, 1.36)	0.06
ESRD			1.97 (1.3, 2.99)	0.0013
Asthma			1.29 (1.02, 1.62)	0.03
COPD			1.22 (0.97, 1.53)	0.09
Depression			1.45 (1.06, 1.98)	0.02

*Note*: Empty cells indicate the variables that were not included in the final model.

Abbreviations: CKD, Chronic kidney disease; COPD, Chronic obstructive pulmonary disease; ESRD, End‐Stage Renal Disease; MI, Myocardial Infarction; SMID, Scottish Index of Multiple Deprivation.

^a^ SBP and DBP were scaled by 10 mmHg; For all of the LTCs, reference category is “no” (yes vs. no).

The results of examining the missingness mechanism in both cohorts are presented in Table S1. In the cohorts from both England and Scotland, the results from imputed data were consistent with a significantly increased association between deprivation and the risk of DFUD. Additionally, long‐term conditions, including PAD, heart failure and atrial fibrillation, increased DFUD risk in both nations. However, country differences emerged: in England, cancer and chronic kidney disease (CKD) histories showed protective effects (HRs: 0.9 [0.8–0.9] and 0.6 [0.6–0.7] respectively), while in Scotland, cancer showed no significant association and CKD increased risk (HR: 1.1). Elevated non‐HDL cholesterol increased DFUD risk by 10% in England only (HR: 1.1 [1.0–1.1]). Smoking increased the risk similarly in both countries (Scotland: HR 1.3 [1.2–1.3]; England: HR 1.4 [1.3–1.5]) (Table 4).

**TABLE 4:** Hazard ratios (HR) of potential risk factors of diabetic foot ulcer development (DFUD) using imputed data.

	CPRD		SDRN‐NDS	
	HR (95% CI)	*p*‐value	HR (95% CI)	*p*‐value
Age at diagnosis (years)	1.04 (1.03, 1.04)	<0.0001***	1.04 (1.04, 1.04)	<0.0001
Sex				
Female versus Male	0.85 (0.81, 0.88)	<0.0001***	0.75 (0.72, 0.79)	<0.0001***
Ethnicity				
Ethnic Minority versus White	0.44 (0.38, 0.50)	<0.0001***	0.72 (0.64, 0.8)	<0.0001*
Index of Multiple deprivation				
Least deprived(reference)	‐	‐	‐	‐
2	1.28 (1.17, 1.41)	<0.0001***	‐	‐
3	1.44 (1.32, 1.57)	<0.0001***	‐	‐
4	1.57 (1.44, 1.72)	<0.0001***	‐	‐
Most deprived	1.98 (1.82, 2.16)	<0.0001***	‐	‐
SIMD				
Most deprived	‐	‐	1.53 (1.41, 1.65)	<0.0001***
2	‐	‐	1.31 (1.21, 1.42)	<0.0001
3	‐	‐	1.24 (1.14, 1.34)	<0.0001***
4	‐	‐	1.17 (1.07, 1.27)	<0.0001
Least deprived (reference)	‐	‐	‐	‐
Smoking				
Current smoker	1.45 (1.36, 1.55)	<0.0001***	1.3 (1.22, 1.38)	<0.0001***
Ex‐smoker	0.92 (0.87, 0.98)	0.01*	0.92 (0.87, 0.97)	0.0025**
Never smoker(reference)	1		‐	‐
Non‐HDL‐C*	1.10 (1.08, 1.13)	<0.0001***		
HbA1c category				
<7%(reference)	‐	‐	‐	‐
7%–8%	0.94 (0.85, 1.03)	0.19	1.15 (1.08, 1.22)	<0.0001***
8%–9%	1.17 (1.03, 1.34)	0.01*	1.23 (1.14, 1.34)	<0.0001***
≥9%	1.40 (1.29, 1.53)	<0.0001	1.48 (1.4, 1.57)	<0.0001
BMI groups				
<25 kg/m^2^	‐	‐	‐	‐
25–30 kg/m^2^	0.92 (0.83, 1.02)	0.13	0.78 (0.72, 0.85)	<0.0001***
≥30 kg/m^2^	1.25 (1.13, 1.38)	<0.0001***	0.8 (0.74, 0.87)	<0.0001***
SBP (mmHg)*	0.95 (0.92, 0.98)	<0.0001***	1.04 (1.03, 1.06)	<0.0001***
DBP (mmHg)**	1.10 (1.08, 1.13)	<0.0001*	0.95 (0.93, 0.98)	<0.0001***
eGFR(ml/min/1.73 m^2^)	0.99 (0.98, 0.99)	<0.0001***	1.01 (1, 1.01)	<0.0001***
*Long‐term conditions*				
Peripheral arterial disease	2.33 (2.10, 2.60)	<0.0001*	2.67 (2.4, 2.97)	<0.0001***
Atrial fibrillation	1.53 (1.41, 1.66)	<0.0001***	1.81 (1.67, 1.97)	<0.0001***
Heart failure	1.72 (1.55, 1.91)	<0.0001***	1.66 (1.43, 1.93)	<0.0001***
Hypertension			1.08 (1.02, 1.15)	0.0083**
Cancer	0.90 (0.82, 0.98)	0.01*		
CKD	0.65 (0.58, 0.72)	<0.0001*	1.11 (1.04, 1.18)	0.0020**
ESRD	1.50 (1.21, 1.86)	<0.0001	1.94 (1.62, 2.32)	<0.0001***
Asthma	1.07 (1.00, 1.15)	0.03*	1.13 (1.03, 1.26)	0.01*
COPD	0.93 (0.84, 1.01)	0.11	1.22 (1.1, 1.36)	0.00013***
Depression	0.91 (0.86, 0.97)	<0.0001***	1.36 (1.21, 1.54)	<0.001**
Dementia			1.51 (1.14, 1.99)	0.0039**
Neuropathy	1.89 (1.49, 2.39)	<0.0001***	2.06 (0.92, 4.6)	0.08
Retinopathy	1.24 (0.99, 1.56)	0.06		

*Note*: *,**,*** based on the level of significancy.

The sensitivity analysis revealed consistent influential factors in both the Post‐QOF Update Cohort and the Post‐SGLT2i Approval Cohort in England. In both cohorts, age at diagnosis, ethnicity and the Index of Multiple Deprivation were significant predictors of DFUD. Elevated pre‐diagnosis HbA1c levels were strongly associated with an increased risk of DFUD in both scenarios (Tables S4 and S5). Additionally, certain long‐term conditions, such as atrial fibrillation and other cardiovascular diseases, showed a substantial impact on the likelihood of developing DFUD. Despite slight variations in specific hazard ratios, the overall trends and significant predictors remained consistent across both cohorts (Table S6).

## 4 CONCLUSIONS

In this study, we investigated risk factors for DFUD in two populations, identifying key sociodemographic and clinical factors influencing incidence rates. Among a combined cohort of 391 790 individuals from England and Scotland, we estimated DFUD incidence rates of 9.0 and 4.4 per 1000 person‐years, respectively. The absolute differences in reported incidence between the two cohorts have been influenced by differing methods of case ascertainment. Notably, the prevalence of MLTCs differed markedly between the cohorts—40.5% in England versus 25.7% in Scotland—despite similar age distributions. This discrepancy suggests that the ascertainment of certain conditions is lower in the Scottish cohort as expected from its reliance on secondary care data for these outcomes. Nevertheless, across both populations, patients with DFUD were more likely to be older, male and of White ethnicity and to have elevated pre‐diagnosis HbA1c levels. We also found that individuals residing in the most deprived areas were at greater risk, and a novel finding of our study is the increased risk of DFUD in those with MLTCs.

The incidence rates of DFUD identified in this study were 3.6 and 1.7 times higher than those reported in a recent study using CPRD data from 2007 to 2017, which found incidence rates of 2.5 and 1.6 per 1000 person‐years for Type 2 and Type 1 diabetes, respectively.^31^ This difference could be attributed to two main factors: methodological differences in the identification of diabetes and DFUD cases—using both primary and secondary care data in England, and secondary care and foot clinic data in Scotland—and the use of different databases and time periods (2007–2017 in the previous study vs. 2000–2021 for England and 2007–2021 for Scotland in this study).

Our findings in two independent contemporary cohorts concerning sociodemographic drivers of DFUD largely confirm those of older.^12^, ^33^, ^34^ As DFUD is a complication of diabetes resulting primarily from long‐term suboptimal glucose control, it is unsurprising that older individuals are at greater risk. The reduced risk of DFUD among individuals from ethnic minorities has been documented in previous studies and may be attributed to lower rates of key conditions that contribute to DFUD development, such as PAD.^20^ For instance, South Asians with diabetes in the United Kingdom have approximately one‐third the risk of developing foot ulcers compared to their European counterparts.^20^ This reduced risk is partially explained by lower levels of PAD, neuropathy, insulin usage and foot deformities in South Asians, which appears to account for about half of the observed reduction in foot ulcer risk.

Our finding of a strong association between socioeconomic deprivation and DFUD risk is in agreement with previous research, including a recent routine healthcare database with newly diagnosed cases of type 2 diabetes using the Health Improvement Network (THIN) data.^35^ Possible explanations for this observation include lifestyle behaviours that are more prevalent in populations with low socioeconomic status, such as reduced physical activity, poor glycaemic control, inadequate blood pressure and lipid management, smoking (also identified as a risk factor in this study) and suboptimal dietary habits^36^ These factors are well‐established drivers of microvascular complications in diabetes, including neuropathy.

Novel findings in this study include a lower incidence of DFUD in ethnic minority groups in England and a decreased risk among overweight and obese individuals in Scotland. Although previous literature has reported a consistently increased risk of DFUD in males compared to females, our study adds value by utilizing two large, community‐based epidemiologic cohorts.^37^ For instance, Zhang et al., in their systematic review and meta‐analysis of foot ulcer disease (FUD), highlighted significant limitations due to high heterogeneity and the fact that 611 226 of the 801 985 participants were from hospital‐based studies, which may not represent the general population.^5^

As expected, glycaemic control above target levels and the presence of most other comorbid conditions were associated with a greater risk of developing DFUD. Poor glycaemic control is a well‐recognized risk factor for DFUD^13^ and elevated pre‐diagnosis HbA1c may also increase the risk of lower extremity amputation in people with DFUD.^38^, ^39^ The presence of MLTCs involving atherosclerotic vascular disease may act as a marker of the risk of DFUD, but MLTCs in general may also increase disease and treatment burden and elevate risk by worsening diabetes control and disease self‐management (including adherence) and mobility.

The incidence of DFUD was lower in the Scottish cohort compared to the English cohort, likely due to differences in case ascertainment methods and potentially other population differences, such as mortality rates from other conditions. Despite these differences, the hazard ratios (HRs) for sociodemographic and clinical covariates were similar across both cohorts, with a few exceptions. For example, HDL cholesterol was inversely associated with DFUD risk in Scotland but not in England, and the association with DBP was reversed, decreasing in Scotland and increasing in England. BMI showed an inverse association with DFUD risk in Scotland, and current smoking was linked to increased DFUD risk only in Scotland.

While key risk factors for DFUD identified in the imputed data were consistent between the two cohorts, such as male sex, glycaemic control, deprivation and MLTC, other factors showed discrepancies. For instance, as non‐HDL cholesterol was a risk factor in Scotland but not England in complete case analysis (Table 3), but not in England or Scotland in imputed analyses (Table 4), a firm conclusion cannot be drawn. Similarly, it is not easy to explain why cancer and chronic kidney disease (CKD) were associated with a lower risk of DFUD in England but not in Scotland. That obesity increased the hazard of DFUD in England but had the opposite effect in Scotland could potentially be attributed to differential missingness. These differences may be partly attributable to the high percentage of missing data in the original datasets (Table S1). Missingness likely influenced the results of the imputation models, despite our efforts to address this issue using machine learning algorithms for data imputation.^40^ However, some discrepancies may reflect differences in the representativeness of the cohorts, with the English cohort being derived from selected primary care practices with over‐representation of less deprived populations, while the Scottish cohort is population‐based.

Our findings will inform future clinical preventative strategies including early diagnosis of diabetes and its complications; intensified management of hyperglycaemia with use of newer treatments such as (e.g. GLP 1 receptor agonists and SGLT 2 inhibitors); intensive, target‐driven management of cardiovascular risk factors (hypertension, hyperlipidaemia, smoking cessation, tailored exercise programmes) and reducing inequality in health by, for example, one‐stop screening services in deprived areas to increase the uptake of NICE recommended annual health checks.^41^, ^42^

Our study has strengths and limitations. Strengths include an incident cohort design using two large, representative UK databases, enabling routine follow‐up from initial diabetes diagnosis to DFUD, death or censoring. We harmonized and pre‐specified our analysis for both cohorts, utilizing linked health and death data to provide comprehensive sociodemographic information and conduct a sensitivity analysis.

Limitations include potential data quality issues, as routine healthcare data are not collected for research purposes, leading to variability in coding of exposure (diabetes), outcome (DFUD) and confounding variables. DFUD may also be variably coded in primary care, though we addressed this with multiple data sources (referrals, primary care and HES).

Additionally, the linked Scottish data lack robust primary care data, limiting the definition of long‐term conditions to hospital records (SMR01). This may partly explain the lower proportion of people with MLTCs in Scotland. Finally, the relationship between care processes and the risk of diabetic foot ulcers (DFUD) was not examined. Although this study did not investigate the impact of care processes on the development of diabetic foot ulcers, future research could explore this aspect more comprehensively. Furthermore, adopting an international perspective may provide valuable insights into variations in care delivery and outcomes across diverse healthcare settings.^17^

In summary, this study robustly identifies risk factors for DFUD in England and Scotland using large, representative routine healthcare databases, including key sociodemographic and clinical variables. Our findings will inform future clinical preventative strategies including early diagnosis of diabetes and its complications; intensified management of hyperglycaemia with the use of newer treatments such (e.g. GLP 1 receptor agonists and SGLT 2 inhibitors); intensive, target‐driven management of cardiovascular risk factors (hypertension, hyperlipidaemia, smoking cessation and tailored exercise programmes), and reducing inequality in health by, for example, one‐stop screening services in deprived areas to increase the uptake of NICE recommended annual health checks.^41^, ^43^

## AUTHOR CONTRIBUTIONS

SG and PH initially drafted the manuscript and subsequently developed it further with JP and EG. KK, JP, EG, CG, FZ, SG, NG, JL, PH, AB and DP made substantial contributions to the conception and design of the work. JL, NG, SG and FZ verified the underlying data. SG and JL conducted data analysis for England and Scotland. MD, JV, FG, BS, GL, LG, ST, DW and SW contributed to the interpretation of the data. All authors were involved in data interpretation, critical review and manuscript revision. All authors had full access to the study data and took full responsibility for the decision to submit the manuscript for publication.

## CONFLICT OF INTEREST STATEMENT

KK has served as a consultant, speaker or has received grants for investigator‐initiated studies from AstraZeneca, Bayer, Novo Nordisk, Sanofi‐Aventis, Servier, Lilly, Merck Sharp & Dohme, Boehringer Ingelheim, Oramed Pharmaceuticals, Pfizer, Roche, Daiichi‐Sankyo, Applied Therapeutics, Embecta and Nestlé Health Science. JV was the National Clinical Director for Diabetes and Obesity at NHS England from April 2013 to September 2023. JP has received non‐financial support as co‐CI of a JDRF‐funded trial (NCT03899402) from AstraZeneca (donation of investigational medicinal product to US site only) and Novo Nordisk [donation of investigational medicinal product to UK site only; supplementary financial support (to mitigate a budget cut during the COVID‐19 pandemic)].

## PEER REVIEW

The peer review history for this article is available at https://www.webofscience.com/api/gateway/wos/peer‐review/10.1111/dom.16519.

## Supporting information


**Data S1:** Supporting Information

## Data Availability

Research data are not shared.
