# Supplementary material for: Risk factors for development of diabetic foot ulcer disease in two large contemporary UK cohorts
Source: Diabetes Obes Metab. 2025 Jun 24;27(9):4782–92. doi: 10.1111/dom.16519 (PMC12326939; doi:10.1111/dom.16519)

# Figure S1: England Study population flow diagram (CPRD-GOLD)


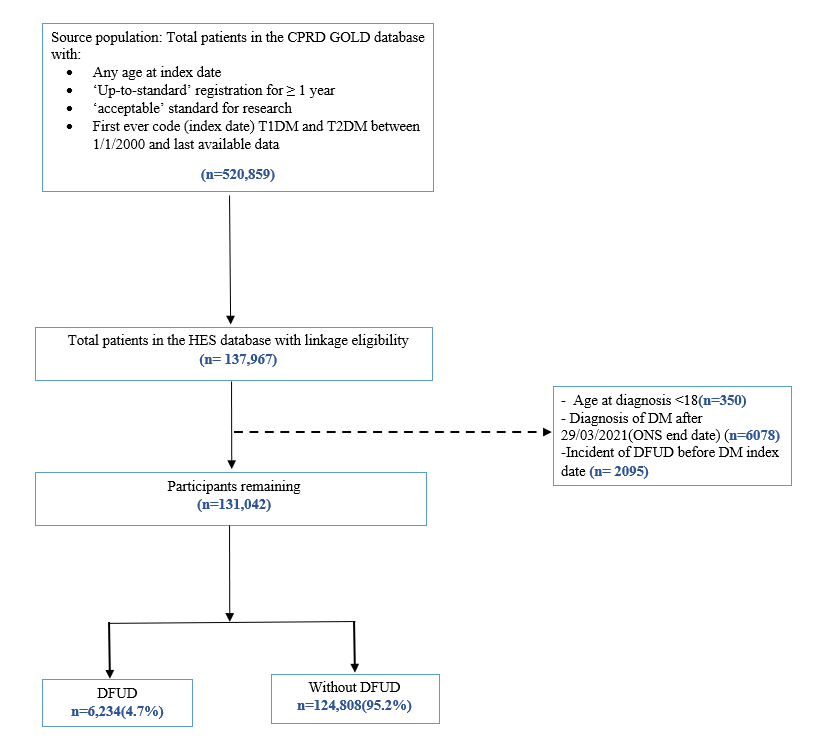


# Figure S2- Scotland study population flow diagram (Scottish Diabetes Research Network-National Diabetes Dataset)


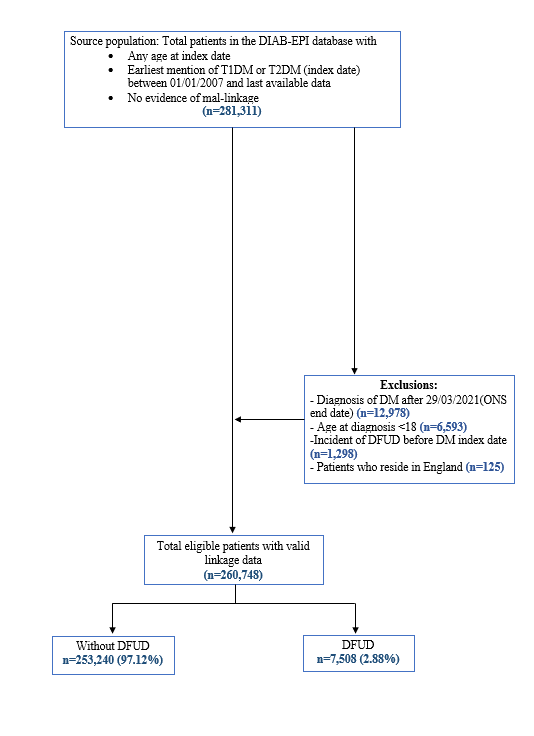


# Figure S3- Study flow and variable measurements time points:


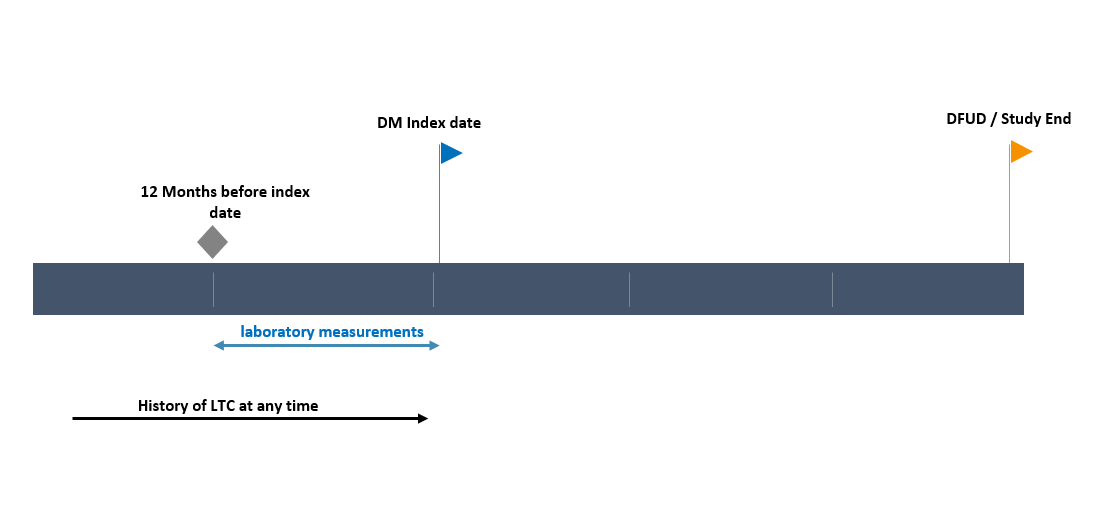


## Table S1- Missing mechanism in incomplete data

|  | **CPRD** | | **SDRN-NDS** | |
| --- | --- | --- | --- | --- |
| Variable | Percentage of missingness | Results of test | Percentage of missingness | Results of test |
| SBP (mmHg) | 36.1% | MAR | 26.8% | MAR |
| DBP (mmHg) | 36.5% | MAR | 26.8% | MAR |
| Non-HDL-C (mmol/L) | 44.5% | MCAR | 46.2% | MAR |
| eGFR (ml/min/1.732m^2^) | 35.1% | MAR | 24.1% | MAR |
| HbA1c (%) | 63.1% | MAR | 44.2% | MAR |
| BMI (kg/m2) | 4.4% | MAR | 47.3% | MAR |
| Ethnicity | 5.0% | MAR | 22.1% | MAR |
| Index of Multiple Deprivation | 0.0% | MCAR | 0.5% | MAR |
| Smoking | 25.2% | MAR | 7.2% | MAR |

## Table S2 – Medication History at index date and 180 days prior

|  | **CPRD** | | | **SDRN-NDS** | | |
| --- | --- | --- | --- | --- | --- | --- |
|  | Total  (N=131,042) | Non-DFUD  (N=124,808) | DFUD  (N=6,234) | Total  (N=260,748) | Non-DFUD  (N=253,240) | DFUD  (N=7,508) |
| Neuropathy Drug | 2,077 (1.6%) | 1,965 (1.6%) | 112 (1.8%) | 36,822(14.1%) | 35,458(14.0%) | 1,364 (18.2%) |
| Thiazolidinediones | 54 (0.0%) | 50 (0.0%) | ** (**%)* | 45 (0.0%) | ** (**%) | ** (**%) |
| SGLT2is | 18 (0.0%) | 17 (0.0%) | ** (**%) | 143 (0.1%) | ** (**%) | **(**%) |
| Insulin | 623 (0.5%) | 591 (0.5%) | 32 (0.5%) | 317 (0.1%) | ** (**%) | ** (**%) |
| Metformin | 15,587(11.9%) | 14,763(11.8%) | 824(13.2%) | 20,564 (7.9%) | 19,939 (7.9%) | 625 (8.3%) |
| GLP-1RAs | ** (**%) | ** (**%) | ** (**%) | 47 (0.0%) | ** (**%) | ** (**%) |
| Glinides | 20 (0.0%) | 18 (0.0%) | ** (**%) | 19 (0.0%) | ** (**%) | ** (**%) |
| DPP-4is | 43 (0.0%) | 39 (0.0%) | 4 (0.1%) | 279 (0.1%) | ** (**%) | ** (**%) |
| Sulphonylureas | 2,567 (2.0%) | 2,368 (1.9%) | 199 (3.2%) | 4,332 (1.7%) | 4,163 (1.6%) | 169 (2.3%) |
| GLDs | 26 (0.0%) | 25 (0.0%) | ** (**%) | 33 (0.0%) | ** (**%) | ** (**%) |

**: Due to data protection rules governing approval to access these data for analysis purposes, any potentially identifiable information (specifically any counts of less than 10) were removed.; SGLT2, sodium-glucose transport protein-2; GLP-1 RA, glucagon-like peptide-1 receptor agonists; DPP-4, dipeptidyl peptidase-4; GLDs, Glucose-Lowering Drugs.

## Table S3 -Crude incidence rate of DFUD (per 1,000 person-years):

|  | **CPRD** | | | **SDRN-NDS** | | |
| --- | --- | --- | --- | --- | --- | --- |
|  | IR (95%CI) | Person-time at risk | p-value* | IR (95%CI) | Person-time at risk | p-value* |
| Total population | 9.0(8.8, 9.2) | 691267.59 |  | 4.38(4.30, 4.47) | 1712288.9 |  |
| Gender |  |  | <0.0001 |  |  | <0.0001 |
| Male | 9.7(9.4, 10.0) | 353517.6 |  | 4.79(4.65, 4.92) | 969093.5 |  |
| Female | 8.3(8.0, 8.6) | 337750.0 |  | 3.86(3.72, 4.0) | 743195.4 |  |
| Age groups |  |  | <0.0001 |  |  | <0.0001 |
| <40 yrs. | 3.7(3.2, 4.1) | 71464.6 |  | 2.56(2.31, 2.79) | 160359.7 |  |
| 40-64 yrs. | 6.9(6.6, 7.2) | 350480.2 |  | 3.28(3.16, 3.39) | 951137.6 |  |
| 65-74 yrs. | 10.4(9.9, 10.8) | 164521.0 |  | 5.16(4.91, 5.4) | 347830.5 |  |
| 75-84 yrs. | 16.3(15.5, 17.2) | 87653.9 |  | 9.18(8.7, 9.67) | 150061.0 |  |
| >85 yrs. | 23.8(21.6, 26.2) | 17147.9 |  | 16.62(14.84, 18.37) | 21361.9 |  |
| Ethnicity |  |  | <0.0001 |  |  | <0.0001 |
| White | 9.9(9.6, 10.1) | 593394.6 |  | 4.58(4.46, 4.7) | 1267639.7 |  |
| South Asian | 4.0(3.2, 4.9) | 23027.0 |  | 1.45(1.09, 1.78) | 45524.6 |  |
| Black | 6.0(4.6, 7.8) | 9334.2 |  | 1.16(0.48, 1.77) | 11216.4 |  |
| Mixed/ Other | 3.0(2.5, 3.6) | 34920.1 |  | 3.13(2.72, 3.54) | 70926.9 |  |
| Quintile of  Index of multiple deprivation (IMD) |  |  | <0.0001 |  |  | - |
| Q1(least deprived) | 6.4(6.0, 7.0) | 131220.5 |  | - | - |  |
| Q2 | 7.8(7.3, 8.3) | 136102.8 |  | - | - |  |
| Q3 | 9.1(8.7, 9.6) | 150776.4 |  | - | - |  |
| Q4 | 9.5(9.0, 10.0) | 138031.9 |  | - | - |  |
| Q5(most deprived) | 12.1(11.5, 12.7) | 134868.1 |  | - | - |  |
| Quintile of  Scottish Index of Multiple Deprivation (SIMD) |  |  | - |  |  | <0.0001 |
| Q1(most deprived) | - | - |  | 5.06(4.85, 5.28) | 409556.8 |  |
| Q2 | - | - |  | 4.51(4.3, 4.72) | 389808.4 |  |
| Q3 | - | - |  | 4.32(4.1, 4.53) | 347477.3 |  |
| Q4 | - | - |  | 4.08(3.86, 4.3) | 312193.1 |  |
| Q5(least deprived) | - | - |  | 3.48(3.25, 3.71) | 243675.1 |  |
| Multiple Long-term Conditions |  |  | <0.0001 |  |  | <0.0001 |
| Yes (≥2) | 12.7(12.3, 13.2) | 254587.6 |  | 7.03(6.76, 7.29) | 386072.9 |  |
| No | 6.8(6.6, 7.1) | 436680.0 |  | 3.62(3.51, 3.72) | 1326215.9 |  |

## Table S4: Baseline Characteristics of Patient Cohort Post-QOF Update (2007-Onwards) in CPRD (England)

|  |  | Total | Non-DFUD | DFUD | **p-value** |
| --- | --- | --- | --- | --- | --- |
|  |  | N=93,416 | N=90,363 | N=3,053 |  |
| Age(yrs.) |  | 58.6 (16.2) | 58.3 (16.2) | 66.4 (14.6) | <0.0001 |
| gender | Male | 45,789 (49.0%) | 44,093 (48.8%) | 1,696 (55.6%) | <0.0001 |
|  | Female | 47,627 (51.0%) | 46,270 (51.2%) | 1,357 (44.4%) |  |
| Ethnicity | White | 77,327 (86.5%) | 74,484 (86.2%) | 2,843 (95.0%) | <0.0001 |
|  | South-Asian | 3,958 (4.4%) | 3,907 (4.5%) | 51 (1.7%) |  |
|  | Black | 1,999 (2.2%) | 1,961 (2.3%) | 38 (1.3%) |  |
|  | Mixed-Other | 6,151 (6.9%) | 6,091 (7.0%) | 60 (2.0%) |  |
| Smoking Status | Current Smoker | 16,781 (22.7%) | 16,191 (22.7%) | 590 (23.3%) | 0.06 |
|  | ex-smoker | 30,795 (41.6%) | 29,799 (41.7%) | 996 (39.4%) |  |
|  | Never Smoker | 26,401 (35.7%) | 25,458 (35.6%) | 943 (37.3%) |  |
| Quintile of Index of Multiple Deprivation | Least Deprived | 17,685 (18.9%) | 17,224 (19.1%) | 461 (15.1%) | <0.0001 |
|  | 2 | 18,165 (19.5%) | 17,673 (19.6%) | 492 (16.1%) |  |
|  | 3 | 19,961 (21.4%) | 19,323 (21.4%) | 638 (20.9%) |  |
|  | 4 | 19,180 (20.5%) | 18,559 (20.5%) | 621 (20.3%) |  |
|  | Most Deprived | 18,392 (19.7%) | 17,552 (19.4%) | 840 (27.5%) |  |
| BMI status |  |  |  |  | <0.0001 |
|  | <25 kg/m^2^ | 11,554(12.3%) | 11,394(12.4%) | 160(10.2%) |  |
|  | 25-30 kg/m^2^ | 26,254(28.1%) | 25,853(28.1%) | 401(25.7%) |  |
|  | ≥ 30 kg/m^2^ | 51,312(54.9%) | 50,372(54.8%) | 940(60.4%) |  |
| BMI (kg/m^2^) |  | 31.2 (27.3-36.1) | 31.2 (27.3-36.0) | 32.8 (28.6-38.4) | <0.0001 |
| SBP (mmHg) |  | 80.7 (11.3) | 80.8 (11.3) | 80.6 (11.8) | 0.52 |
| DBP (mmHg) |  | 136.9 (18.6) | 136.8 (18.6) | 140.5 (19.2) | <0.0001 |
| Creatinine(µmol/L) |  | 79.0 (67.0-92.0) | 79.0 (67.0-92.0) | 82.0 (68.0-98.0) | <0.0001 |
| eGFR(ml/min/1.73m^2^) |  | 77.0 (61.7-90.0) | 77.0 (62.0-90.0) | 74.0 (60.0-90.0) | <0.0001 |
| HbA1c (%) |  | 6.8 (6.3-8.0) | 6.8 (6.3-8.0) | 7.1 (6.5-8.8) | <0.0001 |
| HbA1c(mmol/ml) |  | 51.0 (45.0-64.0) | 51.0 (45.0-63.9) | 54.1 (48.0-73.0) | <0.0001 |
| Non-HDL-C |  | 3.8(3.0- 4.6) | 3.8(3.0- 4.6) | 3.5(2.7-4.4) | <0.0001 |
| Long-term conditions | | | |  |  |
| Atrial Fibrillation |  | 6,489 (6.9%) | 6,007 (6.6%) | 482 (15.8%) | <0.0001 |
| Cancer |  | 9,791 (10.5%) | 9,394 (10.4%) | 397 (13.0%) | <0.0001 |
| COPD |  | 6,801 (7.3%) | 6,428 (7.1%) | 373 (12.2%) | <0.0001 |
| Dementia |  | 1,145 (1.2%) | 1,093 (1.2%) | 52 (1.7%) | 0.01 |
| Depression |  | 23,695 (25.4%) | 22,938 (25.4%) | 757 (24.8%) | 0.46 |
| ESRD |  | 793 (0.8%) | 730 (0.8%) | 63 (2.1%) | <0.0001 |
| Heart Failure |  | 3,373 (3.6%) | 3,100 (3.4%) | 273 (8.9%) | <0.0001 |
| Hypertension |  | 41,403 (44.3%) | 39,535 (43.8%) | 1,868 (61.2%) | <0.0001 |
| MI |  | 5,241 (5.6%) | 4,984 (5.5%) | 257 (8.4%) | <0.0001 |
| Neuropathy |  | 523 (0.6%) | 475 (0.5%) | 48 (1.6%) | <0.0001 |
| Peripheral arterial disease |  | 1,945 (2.1%) | 1,711 (1.9%) | 234 (7.7%) | <0.0001 |
| Retinopathy |  | 782 (0.8%) | 743 (0.8%) | 39 (1.3%) | <0.0001 |
| Stroke |  | 5,922 (6.3%) | 5,576 (6.2%) | 346 (11.3%) | <0.0001 |
| Prevalent Cardiovascular Disease |  | 10,416 (11.2%) | 9,864 (10.9%) | 552 (18.1%) | <0.0001 |
| Multiple long-term conditions |  | 38,467 (41.2%) | 36,662 (40.6%) | 1,805 (59.1%) | <0.0001 |

Data are presented as mean (SD) or median (IQR) for continuous measures, and n (%) for categorical measures. Data are presented as mean (SD) or median (IQR) for continuous measures, and n (%) for categorical measures. Abbreviations: IMD, Index of Multiple Deprivation; BMI, body mass index; SBP, Systolic Blood Pressure; DBP, Diastolic Blood Pressure; eGFR, estimated Glomerular Filtration Rate; HbA1c, glycated haemoglobin; COPD, Chronic obstructive pulmonary disease; CKD, Chronic kidney disease; ESRD, End-Stage Renal Disease; MI, Myocardial Infarction. *Having two or more than two long-term conditions.

## Table S5: Baseline Characteristics of Patient Cohort SGLT2i era (2010-Onwards)

|  |  | **CPRD** | | | | **SDRN-NDS** | | | |
| --- | --- | --- | --- | --- | --- | --- | --- | --- | --- |
|  |  | Total  N=66,247 | Non-DFUD  N=64,476 | DFUD  N=1,771 | p-value | Total  N = 203,003 | Non-DFUD  N = 198,489 | DFUD  N = 4,514 | p-value |
|  |  |  |  |  |  |  |  |  |  |
| Age(yrs.) |  | 58.1 (16.3) | 57.9 (16.2) | 67.0 (14.8) | <0.0001 | 59.2 (14.0) | 59.1 (13.9) | 64.5 (14.0) | <0.0001 |
|  |  |  |  |  | <0.0001 |  |  |  | <0.0001 |
| gender | Male | 32,204 (48.6%) | 31,221 (48.4%) | 983 (55.5%) |  | 115,552 (56.9%) | 112,747 (56.8%) | 2,805 (62.1%) |  |
|  | Female | 34,043 (51.4%) | 33,255 (51.6%) | 788 (44.5%) |  | 87,451 (43.1%) | 85,742 (43.2%) | 1,709 (37.9%) |  |
| Ethnicity |  |  |  |  | <0.0001 |  |  |  | <0.0001 |
|  | White | 53,732 (84.4%) | 52,087 (84.1%) | 1,645 (94.5%) |  | 140,111 (90.2%) | 136,718 (90.1%) | 3,393 (95.2%) |  |
|  | South-Asian | 3,240 (5.1%) | 3,212 (5.2%) | 28 (1.6%) |  | 5,479 (3.5%) | 5,434 (3.6%) | 45 (1.3%) |  |
|  | Black | 1,700 (2.7%) | 1,673 (2.7%) | 27 (1.6%) |  | 1,468 (0.9%) | ** (**%) | ** (**%) |  |
|  | Mixed-Other | 5,027 (7.9%) | 4,987 (8.0%) | 40 (2.3%) |  | 8,217 (5.3%) | 8,100 (5.3%) | 117 (3.3%) |  |
| Smoking Status |  |  |  |  | 0.07 |  |  |  | <0.0001 |
|  | Current Smoker | 12,176 (23.2%) | 11,831 (23.2%) | 345 (23.6%) |  | 40,941 (21.7%) | 39,865 (21.6%) | 1,076 (25.8%) |  |
|  | ex-smoker | 21,902 (41.7%) | 21,335 (41.8%) | 567 (38.9%) |  | 70,553 (37.5%) | 68,965 (37.4%) | 1,588 (38.1%) |  |
|  | Never Smoker | 18,481 (35.2%) | 17,934 (35.1%) | 547 (37.5%) |  | 76,836 (40.8%) | 75,330 (40.9%) | 1,506 (36.1%) |  |
| Quintile of Index of Multiple Deprivation |  |  |  |  | <0.0001 |  |  |  | - |
|  | Least Deprived | 12,693 (19.2%) | 12,426 (19.3%) | 267 (15.1%) |  | - | - | - |  |
|  | 2 | 12,617 (19.1%) | 12,322 (19.1%) | 295 (16.7%) |  | - | - | - |  |
|  | 3 | 14,127 (21.3%) | 13,738 (21.3%) | 389 (22.0%) |  | - | - | - |  |
|  | 4 | 13,641 (20.6%) | 13,298 (20.6%) | 343 (19.4%) |  | - | - | - |  |
|  | Most Deprived | 13,148 (19.9%) | 12,671 (19.7%) | 477 (26.9%) |  | - | - | - |  |
| Quintile of Scottish Index of Multiple Deprivation |  |  |  |  | - |  |  |  | <0.0001 |
|  | Most Deprived | - | - | - |  | 50,039 (24.8%) | 48,754 (24.7%) | 1,285 (28.6%) |  |
|  | 2 | - | - | - |  | 46,757 (23.1%) | 45,716 (23.1%) | 1,041 (23.2%) |  |
|  | 3 | - | - | - |  | 40,916 (20.2%) | 40,036 (20.3%) | 880 (19.6%) |  |
|  | 4 | - | - | - |  | 36,444 (18.0%) | 35,666 (18.0%) | 778 (17.3%) |  |
|  | Least Deprived | - | - | - |  | 28,018 (13.9%) | 27,509 (13.9%) | 509 (11.3%) |  |
| BMI status |  |  |  |  | 0.05 |  |  |  |  |
|  | <25 kg/m^2^ | 8,367(12.6%) | 8,281(12.6%) | 86(9.74%) |  | 8,954 (8.4%) | 8,670 (8.3%) | 284 (13.1%) | <0.0001 |
|  | 25-30 kg/m^2^ | 18,613(28.1%) | 18,366(28.1%) | 247(27.9%) |  | 25,912 (24.4%) | 25,335 (24.4%) | 577 (26.5%) |  |
|  | ≥ 30 kg/m^2^ | 36,190(54.6%) | 35,680(45.6%) | 510(57.7%) |  | 71,234 (67.1%) | 69,920 (67.3%) | 1,314 (60.4%) |  |
| BMI (kg/m^2^) |  | 31.2 (27.3-36.1) | 31.2 (27.2-36.1) | 32.6 (28.6-37.9) | <0.0001 | 32.6 (28.7-37.5) | 32.7 (28.7-37.5) | 31.6 (27.4-36.4) | <0.0001 |
| SBP (mmHg) |  | 80.7 (11.3) | 80.7 (11.2) | 80.5 (11.9) | 0.48 | 137.8 (17.2) | 137.8 (17.1) | 137.8 (18.6) | 0.56 |
| DBP (mmHg) |  | 136.2 (18.3) | 136.0 (18.3) | 139.6 (18.9) | <0.0001 | 80.8 (10.9) | 80.9 (10.9) | 78.7 (11.5) | <0.0001 |
| Creatinine(µmol/L) |  | 77.0 (66.0-90.0) | 77.0 (66.0-90.0) | 80.0 (66.0-97.0) | <0.0001 | 76.0 (66.0-89.0) | 76.0 (66.0-89.0) | 79.0 (67.0-95.0) | <0.0001 |
| eGFR(ml/min/1.73m^2^) |  | 77.0 (62.0-90.0) | 77.2 (62.0-90.0) | 74.0 (60.0-90.0) | <0.0001 | 79.9 (67.4-94.0) | 80.0 (67.5-94.0) | 76.2 (61.5-92.6) | <0.0001 |
| HbA1c (%) |  | 6.8 (6.3-7.8) | 6.8 (6.2-7.7) | 7.0 (6.5-8.4) | <0.0001 | 7.2 (6.6-9.2) | 7.2 (6.6-9.1) | 7.6 (6.7-10.3) | <0.0001 |
| HbA1c(mmol/ml) |  | 50.8 (45.0-61.7) | 50.8 (44.3-61.0) | 53.0 (48.0-68.3) | <0.0001 | 55.0 (49.0-77.0) | 55.0 (49.0-76.0) | 60.0 (50.0-89.0) | <0.0001 |
| Non-HDL-C |  | 3.7(3.0- 4.6) | 3.7(2.7- 4.3) | 3.3(2.7- 4.3) | <0.0001 | 3.9 (3.1-4.7) | 3.9 (3.1-4.8) | 3.6 (2.9-4.6) | <0.0001 |
| Long-term conditions | | | | | | | | | |
| Atrial Fibrillation |  | 4,542 (6.9%) | 4,219 (6.5%) | 323 (18.2%) | <0.0001 | 10,179 (5.0%) | 9,651 (4.9%) | 528 (11.7%) | <0.0001 |
| Cancer |  | 7,051 (10.6%) | 6,783 (10.5%) | 268 (15.1%) | <0.0001 | 19,832 (9.8%) | 19,299 (9.7%) | 533 (11.8%) | <0.0001 |
| CKD |  | 5,618 (8.5%) | 5,301 (8.2%) | 317 (17.9%) | <0.0001 | 48,242 (23.8%) | 46,821 (23.6%) | 1,421 (31.5%) | <0.0001 |
| COPD |  | 5,027 (7.6%) | 4,774 (7.4%) | 253 (14.3%) | <0.0001 | 8,351 (4.1%) | 8,030 (4.0%) | 321 (7.1%) | <0.0001 |
| Dementia |  | 858 (1.3%) | 820 (1.3%) | 38 (2.1%) | 0.70 | 858 (0.4%) | 819 (0.4%) | 39 (0.9%) | <0.0001 |
| Depression |  | 17,286 (26.1%) | 16,831 (26.1%) | 455 (25.7%) | <0.0001 | 6,862 (3.4%) | 6,672 (3.4%) | 190 (4.2%) | 0.002 |
| ESRD |  | 529 (0.8%) | 492 (0.8%) | 37 (2.1%) | <0.0001 | 1,521 (0.7%) | 1,431 (0.7%) | 90 (2.0%) | <0.0001 |
| Heart Failure |  | 2,348 (3.5%) | 2,158 (3.3%) | 190 (10.7%) | <0.0001 | 1,869 (0.9%) | 1,737 (0.9%) | 132 (2.9%) | <0.0001 |
| Hypertension |  | 28,724 (43.4%) | 27,591 (42.8%) | 1,133 (64.0%) | <0.0001 | 34,263 (16.9%) | 33,199 (16.7%) | 1,064 (23.6%) | <0.0001 |
| MI |  | 3,528 (5.3%) | 3,381 (5.2%) | 147 (8.3%) | <0.0001 | 12,073 (5.9%) | 11,726 (5.9%) | 347 (7.7%) | <0.0001 |
| Neuropathy |  | 375 (0.6%) | 342 (0.5%) | 33 (1.9%) | <0.0001 | 53 (0.0%) | ** (**%) | ** (**%) | 0.3 |
| Peripheral arterial disease |  | 1,396 (2.1%) | 1,238 (1.9%) | 158 (8.9%) | <0.0001 | 3,291 (1.6%) | 3,026 (1.5%) | 265 (5.9%) | <0.0001 |
| Retinopathy |  | 560 (0.8%) | 534 (0.8%) | 26 (1.5%) | <0.0001 | 120 (0.1%) | ** (**%) | ** (**%) | 1.00 |
| Stroke |  | 4,133 (6.2%) | 3,916 (6.1%) | 217 (12.3%) | <0.0001 | 8,822 (4.3%) | 8,486 (4.3%) | 336 (7.4%) | <0.0001 |
| Cardiovascular diseases |  | 11,055(16.7%) | 10,477(16.2%) | 578(32.6%) | <0.0001 | 34,946 (17.2%) | 33,713 (17.0%) | 1,233 (27.3%) | <0.0001 |
| Multiple long-term conditions |  | 27,218 (41.1%) | 26,104 (40.5%) | 1,114 (62.9%) | <0.0001 | 52,730 (26.0%) | 50,993 (25.7%) | 1,737 (38.5%) | <0.0001 |

Data are presented as mean (SD) or median (IQR) for continuous measures, and n (%) for categorical measures. Data are presented as mean (SD) or median (IQR) for continuous measures, and n (%) for categorical measures. Abbreviations: IMD, Index of Multiple Deprivation; BMI, body mass index; SBP, Systolic Blood Pressure; DBP, Diastolic Blood Pressure; eGFR, estimated Glomerular Filtration Rate; HbA1c, glycated haemoglobin; COPD, Chronic obstructive pulmonary disease; CKD, Chronic kidney disease; ESRD, End-Stage Renal Disease; MI, Myocardial Infarction. *Having two or more than two long-term conditions. **Censored according to the statistical disclosure control.

|  | Post-QOF Update cohort  (2007-Onwards) | | Post-SGLT2i Approval Cohort | | | |
| --- | --- | --- | --- | --- | --- | --- |
|  | CPRD | | CPRD | | SDRN-NDS | |
|  | HR (95% CI) | p-value | HR (95% CI) | p-value | HR (95% CI) | p-value |
| Age at diagnosis(yrs.) | 1.02(1.01, 1.03) | <0.0001 | 1.03(1.02, 1.03) | <0.0001 | 1.04(1.04, 1.05) | <0.0001 |
| Sex |  |  |  |  |  |  |
| Female vs Male |  |  |  |  | 0.9(0.77, 1.06) | 0.22 |
| Ethnicity |  |  |  |  |  |  |
| Ethnic Minority vs White | 0.43(0.33, 0.57) | <0.0001 | 0.41(0.30, 0.55) | <0.001 | 0.85(0.61, 1.17) | 0.31 |
| Index of Multiple  deprivation |  |  |  |  |  |  |
| Least deprived(reference) | - |  | - |  | - | - |
| 2 | 1.29(1.01, 1.63) | 0.03 | 1.20(0.94, 1.53) | 0.13 | - | - |
| 3 | 1.43(1.14, 1.79) | <0.0001 | 1.38(1.10, 1.73) | <0.0001 | - | - |
| 4 | 1.23(0.97, 1.55) | 0.08 | 1.22(0.96, 1.55) | 0.10 | - | - |
| Most deprived | 2.32(1.88, 2.86) | <0.0001 | 1.99(1.59, 2.47) | <0.0001 | - | - |
| Scottish Index of Multiple  deprivation |  |  |  |  |  |  |
| Most deprived | - | - | - | - | 1.38(1.06, 1.81) | 0.02 |
| 2 | - | - | - | - | 1.17(0.89, 1.55) | 0.26 |
| 3 | - | - | - | - | 1.27(0.96, 1.68) | 0.1 |
| 4 | - | - | - | - | 1.09(0.81, 1.46) | 0.57 |
| Least deprived(reference) | - | - | - | - | - | - |
| Smoking |  |  |  |  |  |  |
| Current smoker |  |  |  |  | 1.46(1.18, 1.81) | 0.00044 |
| Ex-smoker |  |  |  |  | 0.97(0.81, 1.17) | 0.77 |
| Never smoker (reference) |  |  |  |  | - | - |
| HbA1c category |  |  |  |  |  |  |
| <7% | - |  | - |  | - |  |
| 7- 8% | 1.54(1.30, 1.82) | <0.0001 | 1.58(1.32, 1.88) | <0.001 | 1.2(0.98, 1.47) | 0.08 |
| 8- 9% | 2.03(1.62, 2.54) | <0.0001 | 1.87(1.46, 2.40) | <0.001 | 1.38(1.04, 1.83) | 0.03 |
| ≥ 9% | 1.95(1.63, 2.33) | <0.0001 | 1.89(1.56, 2.28) | <0.001 | 1.97(1.61, 2.4) | <0.0001 |
| BMI groups |  |  |  |  |  |  |
| <25kg/m2 (reference) |  |  |  |  | - | - |
| 25-30kg/m2 |  |  |  |  | 0.69(0.53, 0.89) | 0.0044 |
| ≥30kg/m2 |  |  |  |  | 0.67(0.53, 0.85) | 0.0012 |
| DBP (mmHg)** | 1.08(1.04, 1.12) | <0.0001 | 1.06(1.03, 1.11) | <0.0001 | 0.88(0.82, 0.96) | 0.0028 |
| Non-HDL-C(mmol/L) | 0.95(0.90, 1.00) | 0.09 | - | - | 0.92(0.84, 1.01) | 0.07 |
| eGFR(ml/min/1.73m2) |  |  |  |  | 1(1, 1.01) | 0.08 |
|  | | | | |  |  |
| Peripheral arterial disease | 2.07(1.61, 2.65) | <0.0001 | 2.18(1.69, 2.82) | <0.0001 | 2.67(1.98, 3.6) | <0.0001 |
| Atrial fibrillation | 1.72(1.41, 2.09) | <0.0001 | 2.03(1.69, 2.44) | <0.0001 | 1.92(1.53, 2.41) | <0.0001 |
| Heart failure | 1.48(1.16, 1.89) | <0.0001 | - | - | 1.67(1.09, 2.56) | 0.02 |
| CKD | 1.33(1.11, 1.59) | <0.0001 | 1.33(1.10, 1.59) | <0.0001 | 1.2(0.98, 1.47) | 0.08 |
| Dementia | 1.41(0.86, 2.29) | 0.16 | 1.35(0.86, 2.12) | 0.19 |  |  |
| ESRD |  |  |  |  | 2.21(1.36, 3.59) | 0.0014 |
| COPD |  |  |  |  | 1.52(1.17, 1.97) | 0.0015 |

Table S6: Survival Analysis of Factors Associated with Incident DFUD in Two Sensitivity Analysis Scenarios

Empty cells indicate the variables that were not included in the final model.;**SBP and DBP were scaled by 10 mmHg; For all of the LTCs, reference category is “no” (yes vs no); COPD, Chronic obstructive pulmonary disease; CKD, Chronic kidney disease; COPD, Chronic Obstructive Pulmonary Disease; ESRD, End-Stage Renal Disease.

# Table S7- Annual Incidence Rate of DFUD in England during the study period

|  | CPRD | | | | | SDRN-NDS | | | | |
| --- | --- | --- | --- | --- | --- | --- | --- | --- | --- | --- |
| Year | Person-time | Failures | Rate | 95% confidence interval | | Person-time | Failures | Rate* | 95% Confidence Interval | |
| 2000-2003* | 2117.04 | 47 | 22.2 | 16.68 | 29.55 |  |  |  |  |  |
| 2004 | 1968.23 | 40 | 20.32 | 14.91 | 27.71 |  |  |  |  |  |
| 2005 | 2947.58 | 51 | 17.3 | 13.15 | 22.77 |  |  |  |  |  |
| 2006 | 4354.71 | 62 | 14.24 | 11.10 | 18.26 |  |  |  |  |  |
| 2007 | 6694.58 | 87 | 13 | 10.53 | 16.03 | 10216.56 | 81 | 7.93 | 6.32 | 9.78 |
| 2008 | 11442.43 | 161 | 14.07 | 12.06 | 16.42 | 28549.38 | 122 | 4.27 | 3.56 | 5.08 |
| 2009 | 13801.6 | 140 | 10.14 | 8.6 | 11.97 | 47012.50 | 177 | 3.76 | 3.24 | 4.35 |
| 2010 | 25031.35 | 237 | 9.47 | 8.34 | 10.75 | 65517.83 | 221 | 3.37 | 2.95 | 3.84 |
| 2011 | 33218.34 | 474 | 14.27 | 13.04 | 15.61 | 82549.59 | 308 | 3.73 | 3.33 | 4.16 |
| 2012 | 35423.98 | 319 | 9.01 | 8.07 | 10.05 | 99900.79 | 410 | 4.1 | 3.72 | 4.51 |
| 2013 | 55817.25 | 479 | 8.58 | 7.85 | 9.39 | 117013.07 | 422 | 3.61 | 3.27 | 3.96 |
| 2014 | 56061.19 | 405 | 7.22 | 6.55 | 7.96 | 132853.68 | 525 | 3.95 | 3.62 | 4.3 |
| 2015 | 59016.86 | 487 | 8.25 | 7.55 | 9.02 | 147389.62 | 598 | 4.06 | 3.74 | 4.39 |
| 2016 | 59891.22 | 685 | 11.44 | 10.61 | 12.33 | 162833.58 | 677 | 4.16 | 3.85 | 4.48 |
| 2017 | 33292.34 | 802 | 24.09 | 22.48 | 25.82 | 176033.52 | 805 | 4.57 | 4.26 | 4.9 |
| 2018 | 32459.2 | 389 | 11.98 | 10.85 | 13.24 | 187437.10 | 893 | 4.76 | 4.46 | 5.08 |
| 2019 | 34892.92 | 382 | 10.95 | 9.9 | 12.1 | 197934.10 | 1017 | 5.14 | 4.83 | 5.46 |
| 2020 | 71549.89 | 464 | 6.48 | 5.92 | 7.1 | 207186.21 | 946 | 4.57 | 4.28 | 4.86 |
| 2021-2022* | 151286.9 | 522 | 3.45 | 3.17 | 3.76 | 54298.87** | 306 | 5.64 | 5.03 | 6.29 |
| Total | 691267.6 | 6233 | 9.02 | 8.8 | 9.24 | 1712288.91 | 7508 | 4.38 | 4.30 | 4.47 |

*Due to the small number of cases and wide confidence intervals observed in individual years, incident cases over these years were combined. **up to the end of study period, 29/03/2021 in SDRN-NDS.

# Graph S1 - Crude Incidence Rate of DFUD

| 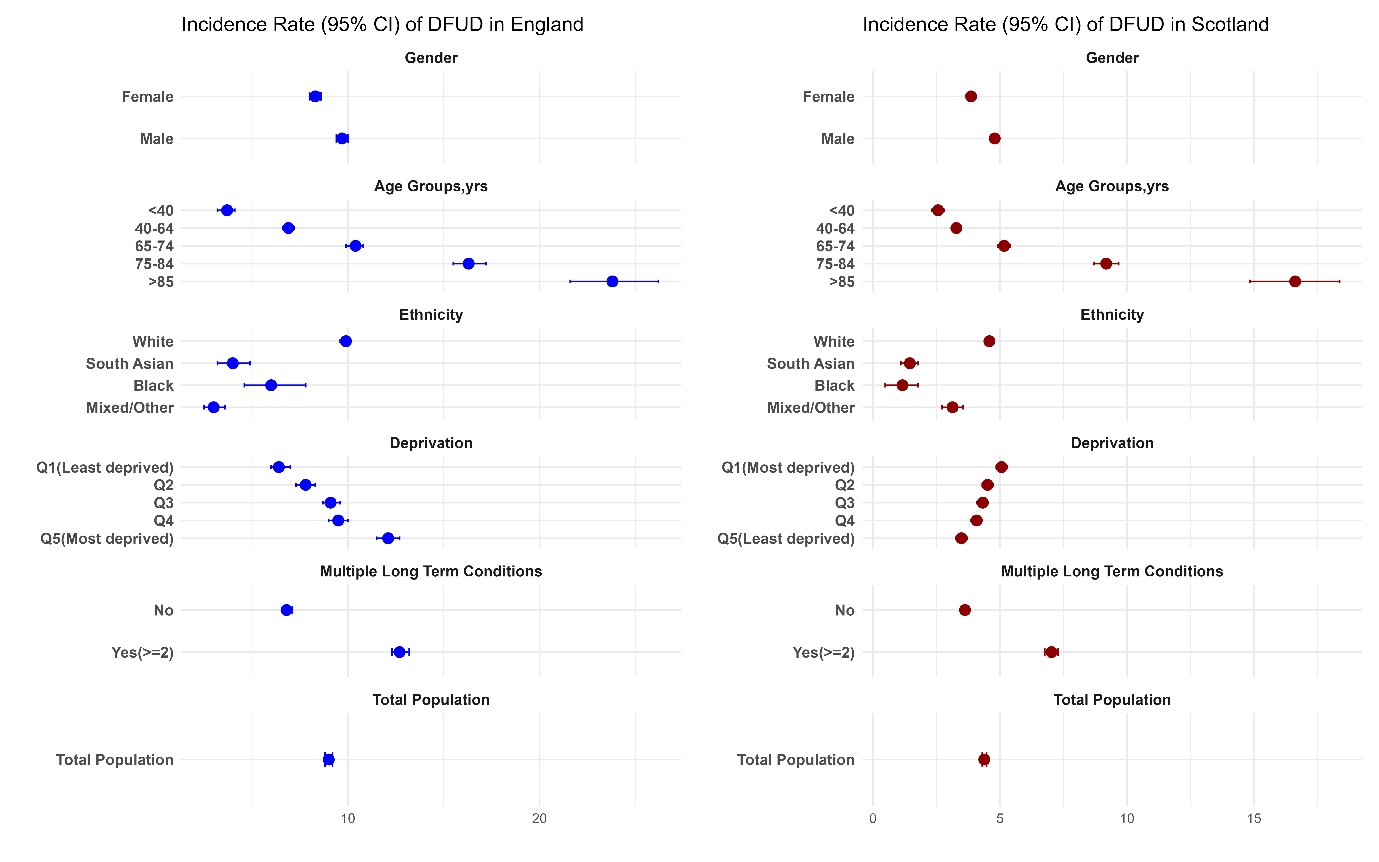 |
| --- |

# Graph S2- Annual Incidence Rate of DFUD in England during the study period


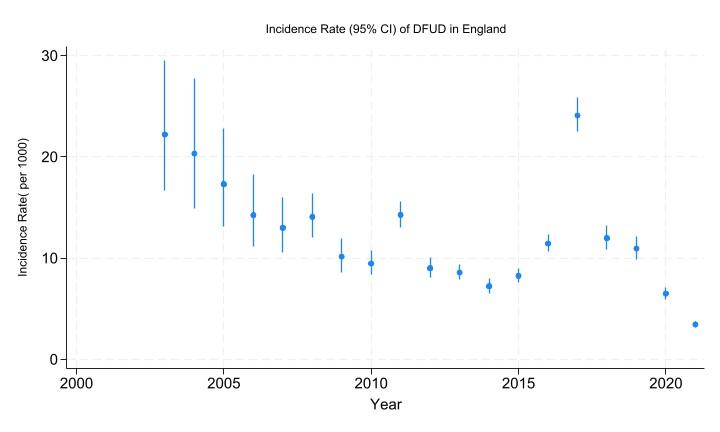


##

## Graph S3: Annual Incidence Rate of DFUD in Scotland during the study period


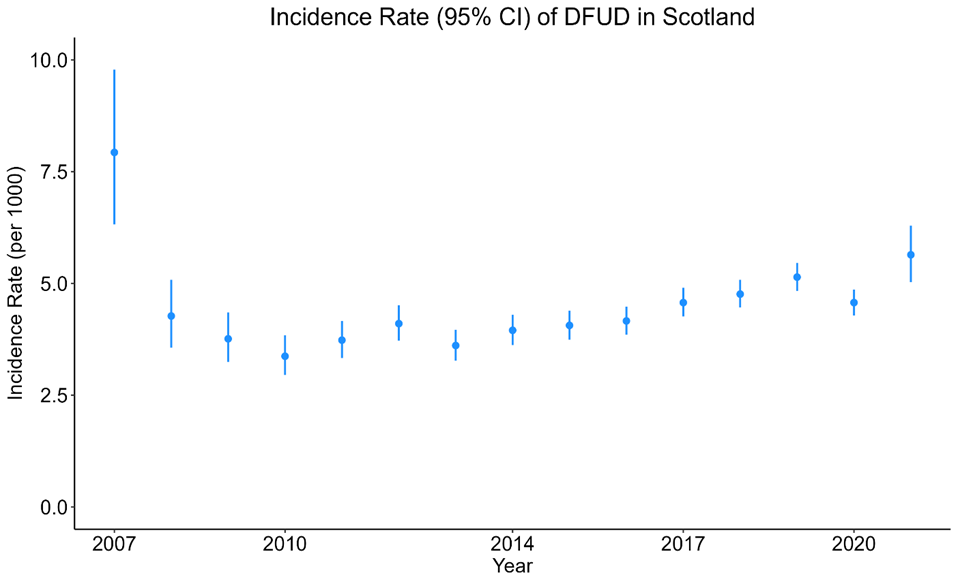

Supplement: Supplementary file 1 — Data S1: Supporting Information [file DOM-27-4782-s001.docx]
